# Mapping the Links Between Celebrity Worship and Subjective Well-Being in Chinese Undergraduates via Network Analysis

**DOI:** 10.3390/bs16010028

**Published:** 2025-12-23

**Authors:** Ke Zhang, Rong Jia, Shiqi Dong, Jingyu Yang, Qing Yang, Liming Zhang

**Affiliations:** 1 School of Education, Huainan Normal University, 238 Dongshan West Road, Tianjia’an District, Huainan 232038, China; zhangk2022email@163.com; 2 School of Psychology, Shaanxi Normal University, 199 Chang’an South Road, Yanta District, Xi’an 710062, China; nulinuligengnuli@163.com (R.J.); ddshiqiii_9@163.com (S.D.); liulian2092@163.com (J.Y.); yangq1110@163.com (Q.Y.); 3 School of Psychology, Zhejiang Normal University, 688 Yingbin Avenue, Wucheng District, Jinhua 321004, China

**Keywords:** celebrity worship, subjective well-being, affect (psychology), college students, China, cross-sectional studies, psychometrics, network analysis

## Abstract

Celebrity worship has become a pervasive phenomenon among Chinese undergraduates, yet its psychological mechanisms remain unclear. This cross-sectional study recruited 1103 Chinese undergraduate students via convenience sampling. Data on celebrity worship and subjective well-being were collected using the Celebrity Attitude Scale (CAS) and the Satisfaction with Life Scale (SWLS). To investigate the internal structure of celebrity worship and its relationship with subjective well-being, a network analysis approach was employed. The resulting networks revealed that 72.33% of possible edges among worship items were non-zero, indicating dense interconnectivity. Entertainment–social behaviors—particularly “obsessed by details of the celebrity’s life”—formed the most central nodes, whereas borderline-pathological beliefs emerged as the pivotal hub when well-being variables were integrated. BP displayed the strongest negative connection with shame and served as the primary bridge linking worship to reduced life satisfaction and heightened negative affect. Bootstrap analyses confirmed robust stability. These findings shift research from a global “total-score” to a “systems” paradigm, highlighting BP cognitions as high-priority targets for cognitive-reappraisal interventions to prevent the escalation from healthy enthusiasm to pathological obsession.

## 1. Introduction

Over the past decade, the global entertainment industry has become deeply inter-twined with social-media platforms, giving rise to a new commercial ecosystem centered on “fan economy” (Liang & Shen, 2016). According to a report by QuestMobile (2024), users born in the 1990s and 2000s account for 41.9% and 31.8%, respectively, of the film and live-entertainment sector—highlighting young consumers’ strong appetite for fandom and its associated spending. Fans are no longer mere “audiences”, but “prosumers” within the entertainment value chain: their emotional involvement, sense of community, and consumption decisions jointly drive the valuation and capital trajectories of idol intellectual property (Liang & Shen, 2016). However, this highly immersive ecosystem, characterized by real-time feedback, also exacerbates the risks associated with extreme fandom. As social media continuously disseminate homogeneous content and community norms strengthen identity fusion, some individuals may develop a borderline-pathological degree of celebrity worship, which can negatively impact subjective well-being, academic or occupational functioning, and social adaptation (Zsila et al., 2018; Zsila et al., 2021). Therefore, adopting a network perspective to investigate the connection mechanisms between celebrity worship and mental health is essential, as it provides a critical theoretical basis for guiding industry regulation and targeted interventions.

According to the Absorption-Addiction Model, celebrity worship can be conceptualized as a continuum that spans from healthy enthusiasm to pathological obsession (McCutcheon et al., 2002; Sansone & Sansone, 2014). At the milder end of the continuum, the entertainment-Social (ES) dimension reflects an interest in celebrities primarily for entertainment and social interaction. While typically considered less pathological than higher levels, some research suggests even ES traits can be associated with maladaptive outcomes. The intermediate level, Intense-Personal (IP), is characterized by compulsive and obsessive feelings toward a celebrity. Finally, the Borderline-Pathological (BP) level represents the most severe form, involving uncontrollable behaviors and fantasies. The model posits that individuals may progress along this continuum as a means of compensating for psychological deficits or establishing a sense of identity (Maltby et al., 2003; McCutcheon et al., 2002).

Furthermore, personal investment is a critical factor—the greater the investment, the more intense the desire for a connection with idols (Branch et al., 2013; T. Y. Chen et al., 2021). According to the Absorption-Addiction Model, high-intensity investment, encompassing sustained expenditure of time, emotional energy, and economic involvement, reinforces an individual’s emotional reliance and psychological attachment to the celebrity (Brooks, 2021). This reliance is rooted, in part, in the self-justification mechanism of cognitive dissonance: to resolve the psychological imbalance between high input and low reciprocal return, individuals often overvalue the idol’s significance and fabricate an illusory “special bond” to rationalize their behavior (Wei & Huang, 2024). On the other hand, the sunk cost effect also drives further investment, as previous psychological and material commitments make disengagement difficult, thereby fostering a desire for deeper connection in pursuit of emotional reward or self-validation. At the neurobiological level, this process may involve the activation of the dopaminergic reward pathway and the development of tolerance, leading individuals to continually seek more intense stimuli to sustain prior levels of pleasure. Consequently, higher investment often correlates with a stronger urge for more intimate—even if unidirectional or imaginary—bonds with the celebrity, resulting in a self-reinforcing cycle of addiction (Maltby et al., 2006). In this process, an initial state of high absorption may progressively intensify, potentially leading to maladaptive or pathological outcomes.

Previous questionnaire-based studies have largely concentrated on the dimensional structure of celebrity worship or the relationship between total scores and psychological well-being (Maltby et al., 2001; Zsila et al., 2021). Zsila et al. have demonstrated that high levels of celebrity worship are significantly and negatively correlated with happiness (Zsila et al., 2021). Moreover, consistent evidence suggests that intense celebrity worship is associated with indicators of maladjustment, including depression, anxiety, and somatization (Maltby et al., 2003; Zsila et al., 2020). However, previous variable-centered research has approached celebrity worship either as a set of independent dimensions or as a global composite score, thereby overlooking two critical issues: which specific symptoms (items) constitute the “core” of pathological worship, and which structural pathways and bridge symptoms connect adaptive forms of celebrity worship (e.g., Entertainment-Social) with maladaptive dimensions (e.g., Borderline-Pathological) within the psychological network. By failing to delineate such granular relationships, these traditional methods are unable to identify pathways of deterioration or clarify the distinct associations different network configurations have with mental health outcomes.

Prior research has extensively mapped the associations between celebrity worship and various indicators of psychological well-being, consistently framing intense celebrity admiration as a maladaptive outcome. Broadly, higher levels of celebrity worship—particularly the Intense-Personal and Borderline-Pathological dimensions—are linked to poorer psychological well-being and elevated negative affect (Brooks, 2021; Maltby et al., 2001). A recent study by Locker and Williams (2025) provides further granularity to these associations by differentiating between positive and negative emotion (Locker & Williams, 2025). Their findings reveal a distinct asymmetry: celebrity worship is significantly and positively correlated with negative emotionality, aligning with the “absorption-addiction” model where obsessive attachment serves as a compensatory mechanism for psychological deficits (McCutcheon et al., 2002). Conversely, celebrity worship shows no significant relationship with positive emotionality, suggesting it contributes little to subjective well-being or positive affective states. While these studies have successfully identified these broad associations using latent variable models, they often rely on sum scores that may obscure the complex interplay between specific emotional states and celebrity worship facets. Consequently, there is a lack of research utilizing network analysis to explicate how specific components of subjective well-being—such as distinct positive and negative emotions—interact with the nuanced dimensions of celebrity worship at the item level. To address these gaps, the present study adopts a network analysis approach. In contrast to latent variable models, network analysis conceptualizes items as interconnected nodes within a dynamic system, enabling the identification of central and bridge nodes (Borsboom & Cramer, 2013; Epskamp et al., 2018). This approach can thus reveal the mechanisms underlying the evolution of celebrity worship and its specific patterns of association with psychological well-being and emotion.

We adopted the Network Theory of Psychopathology as our conceptual rationale (Borsboom et al., 2021). Unlike traditional latent variable models, this approach conceptually frames celebrity worship not as a static trait, but as a dynamic system where specific thoughts and behaviors actively reinforce one another. Accordingly, the present study utilized a cross-sectional design, aiming to explore the internal structure of celebrity worship and its association with subjective well-being via network analysis. Specifically, the study addresses the following research questions and hypotheses: (1) Based on the continuum model (McCutcheon et al., 2002), we expect the Intense-Personal (IP) items to act as bridge nodes connecting the adaptive Entertainment-Social (ES) dimension with the maladaptive Borderline-Pathological (BP) dimension. (2) Drawing on the asymmetry identified by Locker and Williams (2025), we hypothesize that negative emotionality items will exhibit strong, direct edges with Borderline-Pathological nodes (supporting the absorption-addiction model), whereas positive emotionality items will show weak or non-significant connections with the pathological dimensions.

## 2. Materials and Methods

### 2.1. Participants

This survey was conducted between May and June, 2022. Participants were recruited from several comprehensive public universities located in Shaanxi province, China, providing a representative sample of university students in the northwestern region. The survey was hosted on Questionnaire Star (QR), a professional online survey platform capable of generating QR codes for easy distribution. We employed a convenience sampling strategy by disseminating the survey link via QR codes through social media platforms (e.g., WeChat) to university students. The inclusion criteria were: (1) full-time undergraduate students; (2) native Chinese speakers; and (3) voluntary participation. A total of 1200 young adults initially participated. To ensure data quality, the following exclusion criteria were applied: (1) response time less than 120 s; (2) distinct regular response patterns (e.g., straight-lining); and (3) self-reported history of neurological or psychiatric disorders. After applying these criteria, 97 invalid questionnaires were removed, resulting in a final sample of 1103 participants (effective rate = 91.9%). The sample included 612 females and 491 males, with an age range of 19 to 24 years (*M* = 20.61, *SD* = 1.25). Regarding sample size sufficiency, although an a priori power analysis was not conducted, post hoc considerations based on network analysis guidelines suggest the sample size is adequate. Simulation studies for network analysis generally recommend sample sizes exceeding 250 to ensure stable edge estimates (Epskamp et al., 2018). Our sample of 1103 far exceeds this threshold, ensuring the robustness of the network estimation. Approval was obtained from the ethics committee of Shaanxi Normal University. The procedures adhered to the Declaration of Helsinki, and informed consent was obtained from all participants.

### 2.2. Measures

*Celebrity worship:* To assess the levels of celebrity worship, the Celebrity Attitude Scale (CAS) was used (Maltby et al., 2002). This scale includes 23 items scored on a 6-point Likert scale, ranging from 0 (strongly disagree) to 5 (strongly agree). The items are organized into three dimensions: Entertainment-Social (e.g., “I love to talk with others who admire my favorite celebrity.”), Intense–Personal (e.g., “I am obsessed by details of my favorite celebrity’s life.”), Borderline-Pathological (e.g., “News about my favorite celebrity is a pleasant break from a harsh world.”). Higher scores indicate higher levels of celebrity worship. The Cronbach’s α coefficients for the total scale and the three subscales in this study were 0.93, 0.88, 0.83, and 0.66, respectively.

*Subjective well-being:* Subjective well-being was evaluated using the Satisfaction with Life Scale (SWLS) and the International Positive and Negative Affect Schedule Short Form (I-PANAS-SF) (Thompson, 2007; Yun et al., 2019). The SWLS was used to measure individuals’ life satisfaction. This scale includes 5 items (e.g., “I am satisfied with my life”), rated on a 7-point Likert scale (1 = “strongly disagree”, 7 = “strongly agree”). Higher scores suggest greater life satisfaction. The Cronbach’s α coefficient for the SWLS in this study was 0.86. The I-PANAS-SF was used to assess participants’ positive and negative emotions. This scale comprises 10 adjectives describing different affective states, with 5 items for the positive dimension and 5 for the negative dimension. Items were rated on a 5-point scale (1 = “never”, 5 = “always”), with higher scores indicating more intense positive or negative affect. The Cronbach’s α coefficients for the positive and negative dimensions of the I-PANAS-SF in this study were 0.81 and 0.92.

*Socioeconomic status (SES):* Family monthly income was used to represent socioeconomic status. It was measured as a categorical variable on a 7-point scale, with higher scores indicating higher income levels (1 = <2000 RMB; 7 = ≥30,000 RMB).

### 2.3. Data Analysis

To address the research questions proposed, the analysis proceeded in three stages. First, to examine the internal structure and test the hypothesis that Intense-Personal items act as bridges (RQ1), we estimated a network consisting solely of the 23 CAS items and calculated centrality and bridge centrality indices. Second, to investigate the specific associations between celebrity worship dimensions and subjective well-being components (RQ2), we computed a joint network including CAS, SWLS, and I-PANAS-SF items, focusing on the edge weights between Borderline-Pathological nodes and emotionality nodes. Third, as an exploratory step to ensure the robustness of our findings across populations, we conducted a gender comparison using the Network Comparison Test (NCT).

*Covariate Control:* Before estimating the symptom network, we controlled for sociodemographic variables via residualization described by Epskamp (2020). Specifically, for each CAS, SWLS, and I-PANAS-SF item, as well as dimensions of CAS, we established a separate linear regression model with gender, age, and SES entered as predictors. The residuals obtained from these models were standardized and used as the variables in subsequent network estimation. Thus, the network variables represent the unique variance of each item (and dimension) independent of gender, age, and SES.

*Network estimation:* The network model was constructed using R software (version 4.4.2). Based on the standardized residuals, a Pearson correlation matrix was computed. Next, a regularized partial-correlation network was estimated by using the EBICglasso method, with the EBIC hyperparameter set to γ = 0.5. LASSO adds an L1-penalty that shrinks small partial correlations toward zero and sets many of them exactly to zero, yielding more sparse and stable network structures. To some extent, this regularization can ensure robust estimation and control for potential biases such as multicollinearity and overfitting (Xi et al., 2023). The LASSO algorithm generates a series of candidate networks, and the EBIC is then used to select the optimal network. EBIC can better recover the true network structure and reduce false-positive edges (Epskamp & Fried, 2018). By combining LASSO and EBIC, a regularized sparse partial-correlation network can be obtained, which more effectively captures the network structure of relationships among variables. The R packages qgraph (version 1.9.8) and bootnet (version 1.6) were used for estimating and visualizing the network structure. In the network models, each symptom was treated as a node, and the connection between two nodes was defined as an edge. A thicker/weighted edge indicates a stronger statistical association after controlling covariates (gender, age, and family income). Blue lines indicated positive associations and pink lines represented negative associations.

*Centrality indices:* Centrality index was computed using the R packages qgraph. In psychological networks, edges represent statistical associations between variables rather than flows of information between nodes. After regularization, such networks are often not fully connected, and positive and negative edges commonly coexist. Traditional path-based centrality indices typically rely on the assumption of a fully connected network and ignore negative edges, which may overestimate the importance of nodes that have both positive and negative edges. Given that our aim was to identify nodes that exert relatively strong effects on the activation, maintenance, or alleviation of the network, we used expected influence (EI) as our centrality index. EI is defined as the signed sum of the weights of all edges connected to a node (Robinaugh et al., 2016). In the presence of negative edges, EI can provide a more accurate representation of a node’s net impact on the overall network than traditional centrality indices (e.g., betweenness, closeness). Consistent with previous studies, the top 5 nodes based on centrality indices were identified as core nodes (Epskamp et al., 2018; Jones et al., 2021; Tang et al., 2024). Bridge expected influence was calculated using the bridge function in the R packages networktools (version 1.6.0). It is the sum of the signed edge weights connecting a to all nodes outside its own community (Jones et al., 2021). Higher bridge expected influence indicates that a node has stronger overall connections to other communities, and is more likely to function as a bridge that links or transmits effects across communities. In addition, predictability was computed using the R package mgm (version 1.2-15). Predictability reflects the connectivity or association between a node and its adjacent nodes. A higher average predictability indicates a lower degree to which a node can be predicted by other nodes within the network, suggesting the need for direct intervention on the node itself or the exploration of other closely related external variables to control it.

*Stability:* To evaluate the robustness of the estimated network parameters and ensure the reliability of our centrality rankings against sampling variation, we conducted a stability analysis. Specifically, we employed the bootstrapping method to estimate the confidence intervals of edge weights and the stability coefficient (CS-coefficient) for centrality indices (Epskamp et al., 2018). The stability of the network was tested using the R package bootnet, with correlation stability (CS) coefficients calculated for expected influence, bridge expected influence, and edge weights, respectively. Higher CS coefficients indicate greater network stability. Values above 0.25 are considered acceptable, whereas values exceeding 0.5 are preferable (Fang et al., 2025; Watson et al., 2025). CS-coefficient exceeding these thresholds indicates that the order of centrality indices is reliable and unlikely to be observed by chance or significantly altered by sampling variation.

*Network comparison:* The R package Network Comparison Test (NCT; version 2.2.2) was used to examine differences in network invariance, global strength (GS) invariance, centrality invariance and edge weights across networks in different gender models. To address potential disparities in sample sizes between groups, the NCT employs a permutation-based test (1000 permutations). This non-parametric approach provides robust *p*-values without assuming equal sample sizes or normal distributions. The differences in Global Strength are viewed as indicators of overall system connectivity/vulnerability, whereas structural differences suggest distinct symptom-maintenance pathways for each gender. Furthermore, for controlling the expected proportion of false discoveries in the set of edge-difference and centrality invariance tests, *p*-values from the edge-wise and centrality-wise comparisons were adjusted using the Benjamini–Hochberg false discovery rate (FDR) procedure. Together, these procedures allowed us to test gender differences in overall connectivity and network structure while appropriately accounting for multiple comparisons.

## 3. Results

### 3.1. Network of Celebrity Worship

**Figure 1:**
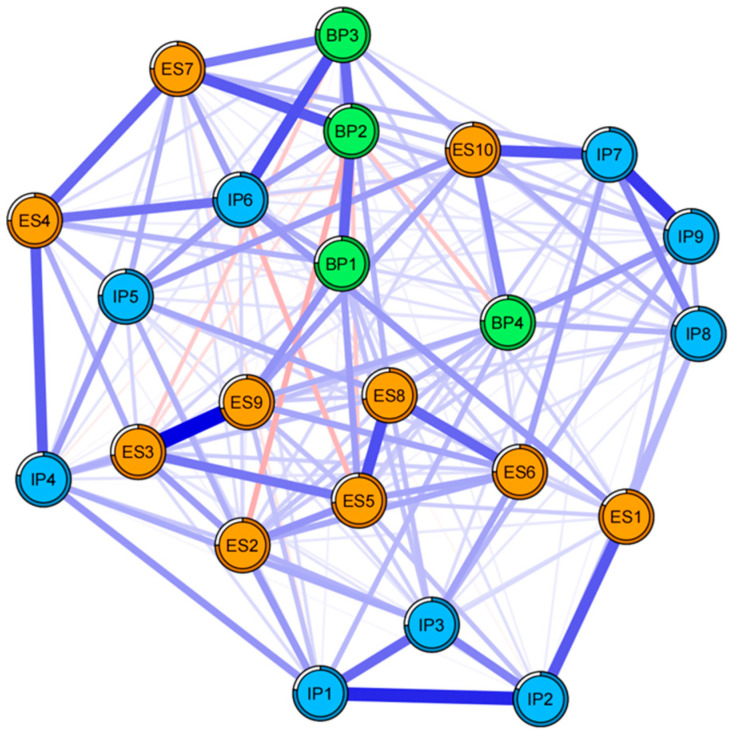
The network structure of young adults’ celebrity worship.

*Network structure:* The celebrity worship network is presented in Figure 1. This network includes 23 nodes, each corresponding to an item from the Celebrity Attitude Scale. Among the 253 possible edges, 183 (72.33%) were non-zero, indicating substantial interconnectedness among the nodes. This high density suggests that celebrity worship functions as a tightly coupled system where activation of one symptom readily spreads to others. The edge weights revealed specific patterns of association. Strong intra-dimensional edges (e.g., ES3–ES9 within the Entertainment-Social dimension; IP1–IP2 within Intense-Personal) highlighted the internal consistency of these sub-constructs. Notably, strong inter-dimensional edges were also observed, such as IP6–BP3 and ES7–BP2. These robust cross-dimension links provide preliminary evidence for the continuum model, illustrating specific pathways through which benign social behaviors may escalate into intense or pathological forms of worship.

*Centrality:* As shown in Table 1, ES9 exhibited the highest expected influence, followed by ES8, BP1, IP3, and ES7. Node predictability, which reflects the degree to which a node can be predicted by other nodes in the network, is also reported in Table 1. Predictability values ranged from 32% to 49%, with overall low values, indicating that the network may be influenced by external factors.

**Figure 2:**
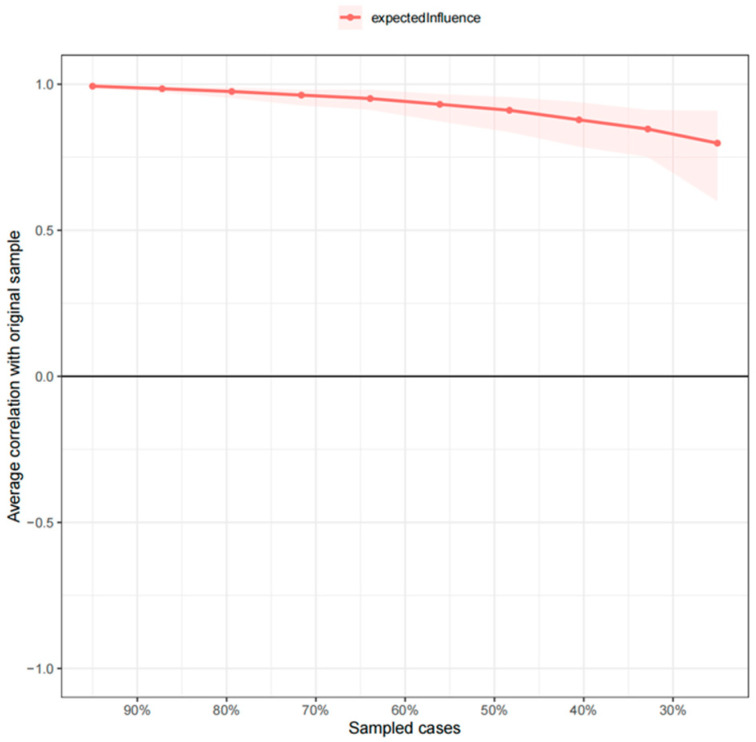
Bootstrap stability test result for expected influence of celebrity worship network.

*Network stability:* The stability of expected influence exceeded the threshold for strong stability, with a correlation stability (CS) coefficient of 0.67 (Figure 2). This indicates that the network structure would remain largely unchanged even if 75% of the participants were excluded from the analysis.

**Figure 3:**
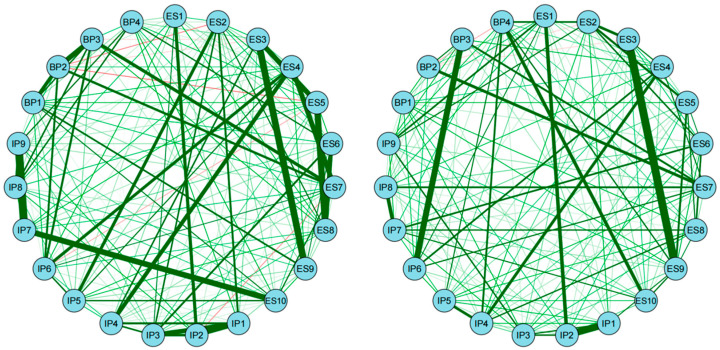
The network structure of female’s (**left**) and male’s (**right**) celebrity worship.

*Network comparison between genders:* The network structures of female and male are shown in Figure 3. Results of network comparison found that there were no significant differences in network structure (*M* = 0.18, *p* = 0.12) nor in global strength between female and male (GS_female_ = 10.96, GS_male_ = 10.54, S = 0.42, *p* = 0.29). Centrality invariance test also showed that node expected influence did not differ across genders. Also, no edge differed significantly across networks. These results suggest that the psychological organization of celebrity worship is structurally invariant across genders, indicating that the interactions between worship symptoms function similarly for both males and females. This implies that despite potential differences in the targets of worship, the fundamental mechanisms maintaining the celebrity worship construct are universal.

### 3.2. Network of Celebrity Worship and Subjective Well-Being

**Figure 4:**
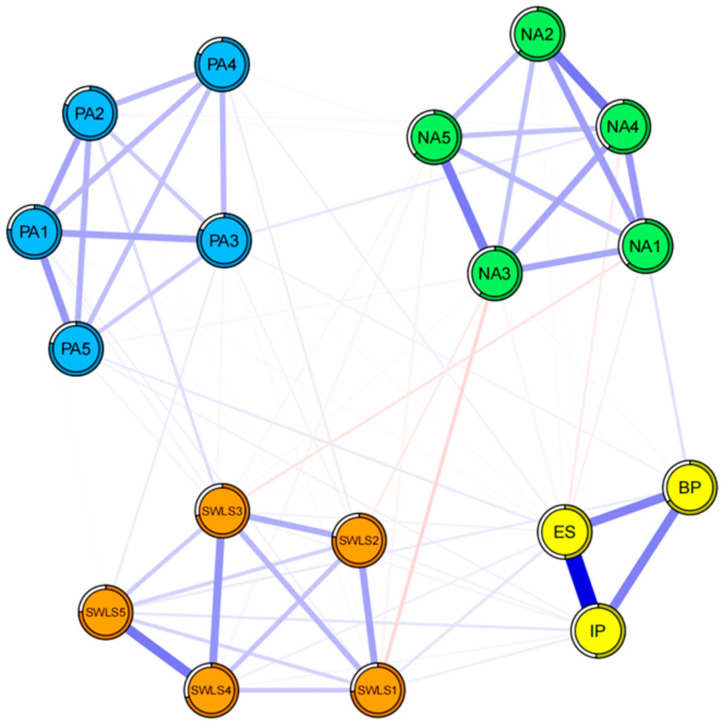
The network structure of young adults’ celebrity worship and subjective well-being.

*Network structure:* This network comprises 18 nodes, each corresponding to a manifestation of celebrity worship and subjective well-being (e.g., life satisfaction, positive affect, and negative affect) (Figure 4). Among the 153 possible edges, 83 (54.25%) were non-zero, indicating substantial interconnectedness among the nodes. Notably, edge strength varied across the network. Within the same community, the edge between ES and IP exhibited the highest weight, followed by those between NA2 and NA4, SWLS4 and SWLS5, NA3 and NA5, and IP and BP. Additionally, regarding connections between celebrity worship manifestations and other constructs, BP–NA4 (shame) represented the strongest positive link between celebrity worship and affect, highlighting BP’s relevance to emotional maladjustment. Subsequently, the edges ES–SWLS1, IP–SWLS5 and BP–SWLS5 connects celebrity worship with the cognitive component of well-being. The edge ES–PA5 further linked the Entertainment–Social dimension to positive affect. This pattern of connectivity illustrates the divergent impact of celebrity worship: while the Entertainment-Social dimension and Intense–Personal dimension are associated with cognitive well-being (Life Satisfaction), the Borderline-Pathological dimension is directly tied to emotional maladjustment (Negative Affect).

**Figure 5:**
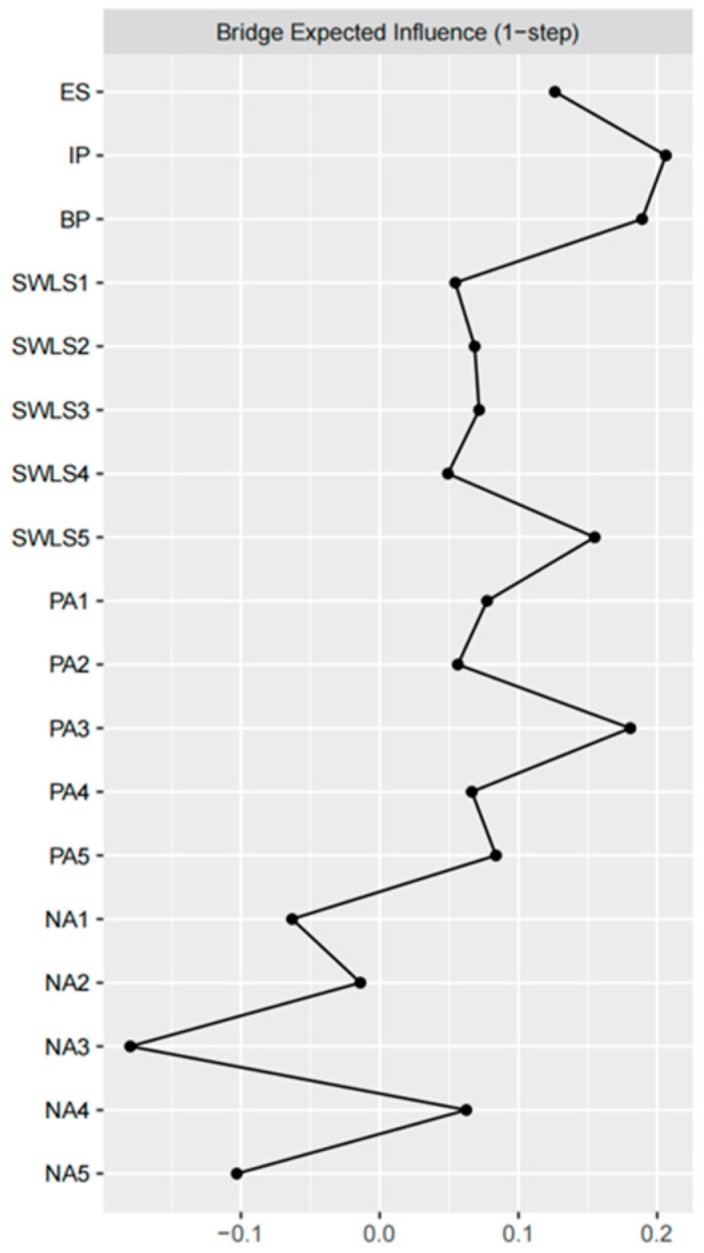
Bridge Expected Influence (1-step). Bars indicate the expected strength with which each node can transmit activation to nodes in other communities (symptom clusters) within one step.

*Centrality:* As shown in Table 2, IP exhibited the highest expected influence, followed by ES, NA4, PA1 and SWLS4. Regarding bridge expected influence, IP had the highest value, followed by BP, PA3, SWLS5, and PA5 (Figure 5). Given the nature of expected influence and bridge expected influence, operationally, intervening on the central node (e.g., IP) or the bridge (BP) can propagate change to adjacent nodes, reducing activation of the sub-system (e.g., NA4: shame). Node predictability, which reflects the degree to which a node can be predicted by other nodes in the network, is also reported in Table 2. Predictability values ranged from 33% to 75%, with generally medium values.

**Figure 6:**
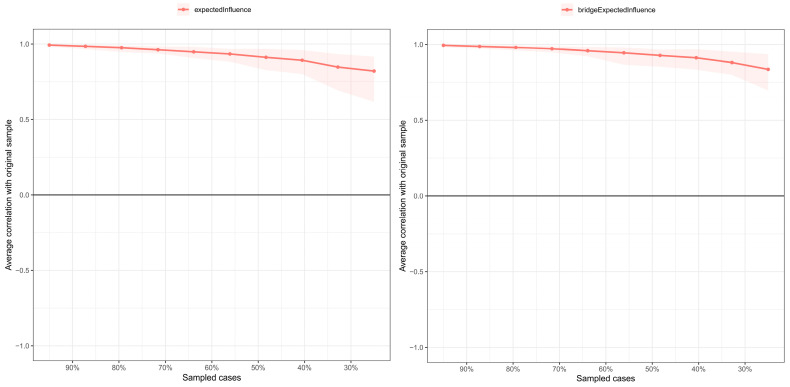
The stability of centrality (**left**) and bridge centrality indices (**right**).

*Network stability:* The stability of both expected influence and bridge expected influence exceeded the threshold for strong stability, with correlation stability (CS) coefficients of 0.67 and 0.75 (Figure 6), respectively. This indicates that the network structure would remain largely unchanged even if 75% of the participants were excluded from the analysis.

**Figure 7:**
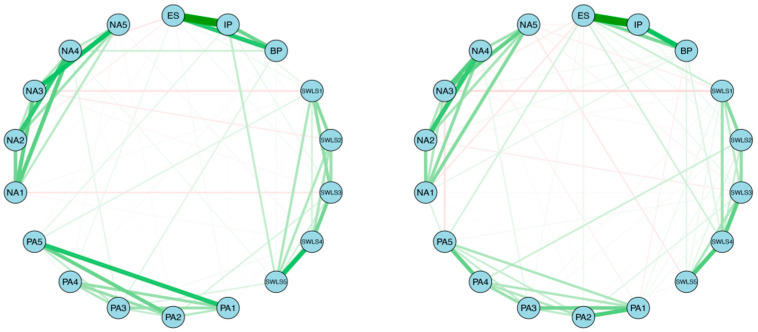
The network structure of female’s (**left**) and male’s (**right**) celebrity worship and subjective well-being.

*Network comparison between genders:* The network structures of female and male are shown in Figure 7. The Network Comparison Test found significant differences in network structure (*M* = 0.23, *p* < 0.05), not in global strength between female and male (GS_female_ = 8.20, GS_male_ = 8.44, S = 0.24, *p* = 0.33). However, centrality invariance test also showed that expected influence of SWLS5 was higher in female’s network than in male’s network. Also, there were significant differences between two networks in edge weights. The edges between BP and SWLS4 and between PA4 and PA5 were significantly stronger in the male network than in the female network (*p* < 0.05).

## 4. Discussion

The celebrity-worship network exhibited moderate density, with 183 of the 253 possible edges (72.33%) being non-zero. This network provides a “systems paradigm” perspective. Rather than assuming a single latent trait, the systems paradigm conceives items as nodes that co-activate and maintain each other; central and bridge nodes become priority targets for preventive and clinical intervention. In other words, celebrity worship can be understood as a connected system in which certain components may trigger or reinforce others, forming a reinforcing cycle. This shift from a traditional “total-score paradigm” to a “systems paradigm” enables the identification of specific cognitions or behaviors that act as catalysts, propelling enthusiasm toward pathological attachment. In addition, this study also highlights a potential pathway from benign engagement with celebrities to more problematic forms of celebrity worship that can negatively impact psychological well-being. From a broader social and cultural perspective, these findings inform regulation of fan economy practices and the design of university preventive programs focused on BP cognitions and shame (NA4).

With respect to the first research question (RQ1) regarding the internal structure and bridge pathways, the analysis yielded a moderately dense network, reflecting robust interconnectivity among the symptom components. Consistent with our hypothesis derived from the continuum model, the network topology revealed a hierarchical progression. The Entertainment-Social (ES) dimension—anchored by highly influential nodes like ES9 (“Enjoy celebrity in groups”) and ES8 (“Celebrity’s news providing entertainment”)—served as the primary activation point. The high density of the network confirms that celebrity worship is a highly cohesive construct. The analysis of edge weights revealed that while the dimensions (ES, IP, BP) form distinct clusters, they are bridged by specific strong connections (e.g., IP6-BP3). This finding is crucial as it uncovers the “micro-mechanisms” of the absorption-addiction process: it is not just a general progression, but specific symptoms—such as “Feel like dying if celebrity dies”—that directly trigger pathological thoughts like “Spend thousands on possession”, thereby facilitating the transition from enthusiasm to obsession.

The identification of ES9, ES8 and the BP cluster as principal hubs can be interpreted through the lenses of Uses-and-Gratifications Theory (Lin & Chu, 2021; Papacharissi & Mendelson, 2007) and Parasocial Relationship Theory (Gong & Huang, 2022; Spitzberg & Cupach, 2008). Specifically, ES1 likely operates as the network’s primary ‘traffic inlet’ because it satisfies fundamental high-frequency needs for diversion and social interaction, offering immediate, low-cost hedonic rewards. In contrast, the centrality of the BP cluster reflects its role as an ‘affective amplifier.’ Consistent with parasocial theory, fans may redirect attachment needs toward the celebrity to compensate for real-life deficits (Widiastuti et al., 2019). The BP items (e.g., rescue fantasies) thus serve to convert the initial informational inputs from ES9 and ES8 into deep emotional attachment and identity fusion. Together, these findings clarify the mechanism behind the continuum model (RQ1): ES9 and ES8 supply the initial engagement via gratification seeking, which the BP cluster then transforms into pathological absorption through compensatory bonding (Brown, 2015; O. Chen et al., 2022; Stever, 2017).

Turning to the second research question (RQ2) regarding the association with well-being, our joint network analysis supported the hypothesized asymmetry between positive and negative emotionality. The most robust negative association in the integrated network linked Borderline-Pathological beliefs (BP) with the shame item of negative affect (NA4) (Maltby & Day, 2017). This pattern aligns with the self-psychology perspective, which posits that extreme idol identification blurs the boundaries between self and other. Consequently, fans internalize perceived discrepancies between their own identity and the idealized image of the celebrity. As this gap widens, self-focused negative emotions—particularly shame—are amplified, manifesting in statements such as “I should be more like my idol” or “I am inadequate compared to my idol”. Thus, BP functions as a cognitive amplifier, converting unrealistic fusion into recurrent self-devaluation (Reeves et al., 2012).

Bootstrap stability analyses yielded correlation stability (CS) coefficients of ≥0.67 for both expected influence and bridge expected influence, well above the preferable threshold of 0.50. This indicates that the estimated network topology is highly robust to sampling variability. These findings support the psychometric integrity of the Celebrity Attitude Scale in a Chinese university sample and justify its use as a reliable instrument for assessing the fine-grained structure of celebrity worship. At the same time, node predictability ranged from 32% to 49%, leaving more than 30% of the variance unexplained by within-network connections. This moderate level of predictability suggests that external factors—such as personality traits (e.g., neuroticism, narcissism) and perceived social support—play a meaningful role in shaping both the severity of celebrity worship and its downstream effects on well-being. Future studies should therefore integrate these extra-network variables into a multilayer framework to account for the residual variance and to refine causal inferences (Fried et al., 2018).

Indeed, while narcissistic traits generally predispose individuals toward the allure of fame, the nature of this association is nuanced. Recent findings by Locker and Williams (2025) illuminate this distinction: they observed that vulnerable narcissism co-occurs with negative affectivity and elevated Celebrity Attitude Scale (CAS) scores. In contrast, grandiose narcissism aligns with positive affectivity and shows no significant relation to celebrity worship. This pattern suggests that negative emotionality serves as a critical bridge, channeling vulnerable traits into maladaptive celebrity attachment. Reinforcing this perspective, Maltby et al. (2006) identified a specific link between neuroticism and the ‘Intense-personal’ dimension of celebrity worship. Their work indicates that individuals exhibiting neurotic traits—such as anxiety, depression, and vulnerability—are prone to obsessive, compulsive attachments, implying that intense celebrity worship is often a manifestation of underlying emotional instability rather than simple entertainment seeking. Consequently, future studies should integrate these constructs into a multilayer framework. By explicitly modeling the interplay between stable personality traits (e.g., vulnerable narcissism and neuroticism), affective states, and behavioral outcomes (celebrity worship), such an approach would better account for residual variance and refine causal inferences regarding the psychopathology of fandom (Fried et al., 2018).

Regarding gender differences, the NCT results revealed a nuanced pattern: while the overall system connectivity (global strength) was similar across genders, the specific structural pathways differed significantly (van Borkulo et al., 2014). First, the higher expected influence of SWLS5 (“no regrets”) in the female network suggests that for women, the sense of life acceptance serves as a pivotal hub, regulating the interaction between worship and well-being more strongly than in men. This aligns with findings that women often employ more emotion-focused coping strategies, such as positive reframing or acceptance, to maintain subjective well-being (Matud, 2004; Tamres et al., 2002). Second, and perhaps most notably, the edge between Borderline-Pathological (BP) worship and SWLS4 (“gotten important things”) was significantly stronger in males. This implies a gender-specific vulnerability: for men, pathological absorption is more intimately tied to their sense of life achievement. This supports the compensatory hypothesis specifically in males—where pathological worship may arise from, or significantly impact, the evaluation of one’s life accomplishments (Schwartz & Rubel Lifschitz, 2006). Consequently, while interventions can target general mechanisms for both genders, practitioners working with male clients should specifically address the link between obsession and perceived life success/failure, whereas for female clients, focusing on global life acceptance (SWLS5) may be more effective.

This study also has several limitations. First, the cross-sectional design precludes any causal inferences. The observed network merely reflects contemporaneous associations between celebrity-worship items and well-being indicators, leaving the temporal ordering of activation unresolved. Second, all data were collected via self-report questionnaires, making the results susceptible to social-desirability bias and shared-method variance. Third, the sample was restricted to Chinese university students aged 19–24 years. Although this enhances internal validity, it limits the generalizability of the findings to older adults, non-student populations, or cultural contexts with different idol ecologies. Fourth, the current network model focuses exclusively on the internal interplay between celebrity worship and subjective well-being. Consequently, it represents a relatively simplified system that does not account for external factors which may influence these pathways. Future research should incorporate these external variables into the network to provide a more comprehensive understanding of the transition from healthy to pathological worship. Specifically, the sample was restricted to university students, who typically exhibit higher digital literacy and social media engagement than the general population. Furthermore, the organized fan culture (e.g., data labor, voting) in China may differ from the more individualistic fan behaviors in Western contexts (Morgan et al., 2024; Zhang & Negus, 2020), potentially influencing the subjective well-being network structure. Therefore, caution should be exercised when generalizing these findings to other age groups or cultural contexts. Finally, although we controlled for gender, age, and socioeconomic status, other potential confounding variables—such as field of study and social media use—were not assessed, suggesting that future research should incorporate these factors to yield a more comprehensive understanding of the symptom network.

## 5. Conclusions

Utilizing network analysis in a large sample of Chinese undergraduates, the present study mapped the fine-grained topology of celebrity worship and its coupling with subjective well-being. Three key findings emerged. Firstly, the entertainment-social (ES) dimension—especially the item “obsessed by details of the celebrity’s life”—constituted the most central node within the celebrity-worship network, whereas the borderline-pathological (BP) cluster became the pivotal hub once well-being indicators were integrated. Secondly, BP exhibited the strongest negative edge with shame and served as the primary bridge linking idol worship to reduced life satisfaction and heightened negative affect, underscoring its clinical salience. Thirdly, stability coefficients exceeded 0.50, attesting to the robustness of the Celebrity Attitude Scale within the Chinese context. Collectively, these results shift celebrity-worship research from a “total-score” to a “systems” paradigm and identify BP as a high-priority target for cognitive-reappraisal and reality-testing interventions. In practice, universities could incorporate brief screening (e.g., CAS and I-PANAS-SF) within routine counseling intake to identify students at elevated risk. They could also offer group interventions for emotional regulation targeting shame (NA4) and BP beliefs in psychological counseling center. In student clubs/associations, psychoeducation on media consumption and community norms should also be emphasized. By integrating screening, prevention, and intervention, it may be possible to prevent normative fandom from escalating into maladaptive celebrity worship and ultimately protect students’ psychological well-being.

## Figures and Tables

**Table 1:** Descriptions and central indices of young adults’ celebrity worship network nodes.

Items	Item Content Abbreviation	*M* (*SD*)	Expected Influence	Predictability
ES1	Escape from problems	3.52 (1.12)	0.81	0.32
ES2	Enjoy celebrity media	3.70 (0.98)	0.92	0.44
ES3	Talk with other admirers	3.77 (0.88)	0.90	0.47
ES4	Share celebrity misfortune	3.48 (1.04)	0.98	0.44
ES5	Enjoy celebrity’s life story	3.74 (1.02)	0.91	0.46
ES6	Enjoy being with other fans	3.68 (1.03)	0.95	0.44
ES7	Feel failure when celebrity fails	3.43 (1.10)	0.99	0.43
ES8	Celebrity’s news providing entertainment	3.74 (1.07)	1.05	0.47
ES9	Enjoy celebrity in groups	3.66 (0.95)	1.06	0.49
ES10	Discuss celebrity’s actions	3.63 (1.08)	0.95	0.42
IP1	Being biggest fan	3.32 (1.08)	0.89	0.40
IP2	Special bond with celebrity	3.42 (1.08)	0.78	0.33
IP3	Obsessed with details	3.63 (0.99)	1.02	0.44
IP4	Share good fortune	3.47 (1.10)	0.88	0.40
IP5	Celebrity’s success as mine	3.63 (1.05)	0.98	0.42
IP6	Feel like dying if celebrity dies	3.24 (1.14)	0.94	0.42
IP7	Keepsakes of celebrity	3.63 (1.15)	0.90	0.40
IP8	Celebrity as soulmate	3.63 (1.05)	0.89	0.36
IP9	Frequent thoughts of celebrity	3.61 (1.07)	0.90	0.37
BP1	Compelled to learn habits	3.50 (1.12)	1.03	0.43
BP2	Do illegal favor for celebrity	3.07 (1.32)	0.55	0.30
BP3	Spend thousands on possession	3.26 (1.18)	0.88	0.38
BP4	Celebrity news as escape	3.55 (1.14)	0.93	0.42

**Notes:** ES: Entertainment-Social dimension of Celebrity Attitude scale; IP: Intense–Personal dimension; BP: Borderline-Pathological dimension. The means and standard deviations reported in the table were calculated from the raw data before residual adjustment.

**Table 2:** Descriptions and central indices of nodes in the network of celebrity worship and subjective well-being.

Items	Item Content Abbreviation	*M* (*SD*)	Expected Influence	Predictability
ES	-	3.63 (0.71)	1.00	0.75
IP	-	3.51 (0.72)	1.08	0.74
BP	-	4.16 (1.25)	0.77	0.56
SWLS1	Close to ideal	3.63 (0.71)	0.66	0.45
SWLS2	Excellent conditions	3.51 (0.72)	0.71	0.40
SWLS3	Satisfaction	4.24 (1.30)	0.80	0.48
SWLS4	Got important things I want	4.17 (1.37)	0.88	0.50
SWLS5	Change nothing	3.90 (1.50)	0.79	0.43
PA1	Determined	3.95 (0.88)	0.91	0.42
PA2	Attentive	3.98 (0.89)	0.73	0.35
PA3	Alert	3.77 (0.91)	0.80	0.33
PA4	Inspired	3.96 (0.88)	0.71	0.33
PA5	Active	4.05 (0.88)	0.77	0.38
NA1	Afraid	2.87 (1.27)	0.77	0.61
NA2	Nervous	2.95 (1.20)	0.84	0.61
NA3	Upset	2.85 (1.23)	0.69	0.64
NA4	Ashamed	2.89 (1.23)	0.97	0.64
NA5	Hostile	2.86 (1.23)	0.69	0.59

**Notes:** ES: Entertainment-Social dimension of Celebrity Attitude scale; IP: Intense–Personal dimension; BP: Borderline-Pathological dimension; SWLS: Satisfaction With Life Scale; PA: positive dimension of International Positive and Negative Affect Schedule Short Form; NA: negative dimension of International Positive and Negative Affect Schedule Short Form. The means and standard deviations reported in the table were calculated from the raw data before residual adjustment.

## Data Availability

The original contributions presented in this study are included in the article/Supplementary Material. Further inquiries can be directed to the corresponding author.

## Supplementary Materials

The following supporting information can be downloaded at: https://www.mdpi.com/article/10.3390/bs16010028/s1.
